# Making Fun of Us

**Published:** 1873-04

**Authors:** 


### MAKING FUN OF US.

WE try to write soberly sometimes, but our wisest sayings are turned into ridicule, yet it must be remembered that a really hearty laugh is worth a peck of pills. Here is what the *Ladies' Repository* says: "It is much easier to take care of the health than it is to recover it. Those are the words of Hall's JOURNAL OF HEALTH, and we perceive that they are being attended to by our youth of both sexes. The ladies have placed another feather in their hats since the cold weather began, and the gentlemen are wearing warmer and heavier sleeve-buttons."

